# Death of Hon. Jonathan Hoyt, M. D.

**Published:** 1851-01

**Authors:** 


*Death of Hon. Jonathan Hoyt, M. D.*—Died, on the 25th Nov. 1850 Hon. JONATHAN HOYT, M. D., aged about sixty years.

The late Dr. Hoyt was an old resident of this county, having been a practicing physician in the town of Aurora for nearly forty years He was a man of eccentric habits, but was much esteemed as a practitioner in the part of the county in which he resided.

He was, for several years, one of the Judges of the county.

His death occurred suddenly. He was walking along Main-st., conversing with a friend, and apparently in perfect health, when he fell upon the side walk, and died almost instantly.

